# Effect of Ions and Sequence Variants on the Antagonist Binding Properties of the Histamine H_1_ Receptor

**DOI:** 10.3390/ijms23031420

**Published:** 2022-01-26

**Authors:** Marcus Conrad, Christian A. Söldner, Heinrich Sticht

**Affiliations:** 1 Division of Bioinformatics, Institute of Biochemistry, Friedrich-Alexander-University Erlangen-Nürnberg (FAU), 91054 Erlangen, Germany; mar.conrad@fau.de (M.C.); christian.soeldner@gmx.de (C.A.S.); 2 Erlangen National High Performance Computing Center (NHR@FAU), Friedrich-Alexander-University Erlangen-Nürnberg (FAU), 91058 Erlangen, Germany

**Keywords:** receptor-ligand interactions, G protein-coupled receptors (GPCRs), molecular dynamics simulations, metadynamics, sodium binding, sodium pocket, phosphate, doxepin, allosteric modulator, sequence variants

## Abstract

The histamine H_1_ receptor (H_1_R) is a G protein-coupled receptor (GPCR) and represents a main target in the treatment of allergic reactions as well as inflammatory reactions and depressions. Although the overall effect of antagonists on H_1_ function has been extensively investigated, rather little is known about the potential modulatory effect of ions or sequence variants on antagonist binding. We investigated the dynamics of a phosphate ion present in the crystal structure and of a sodium ion, for which we determined the position in the allosteric pocket by metadynamics simulations. Both types of ions exhibit significant dynamics within their binding site; however, some key contacts remain stable over the simulation time, which might be exploited to develop more potent drugs targeting these sites. The dynamics of the ions is almost unaffected by the presence or absence of doxepin, as also reflected in their small effect (less than 1 kcal·mol^−1^) on doxepin binding affinity. We also examined the effect of four H_1_R sequence variants observed in the human population on doxepin binding. These variants cause a reduction in doxepin affinity of up to 2.5 kcal·mol^−1^, indicating that personalized medical treatments that take into account individual mutation patterns could increase precision in the dosage of GPCR-targeting drugs.

## 1. Introduction

G protein-coupled receptors are a large protein family with more than 800 members in humans [1,2]. Most GPCRs are regulated by extracellular ligands, which modulate the interaction between GPCRs and their intracellular binding partners, thereby triggering cellular signaling cascades [3]. GPCRs play a key role in many physiological processes like neurotransmission [4], allergic reactions [5], or cardiac function [6]. Due to their medical importance, GPCRs represent important drug targets, and more than 30% of the currently approved drugs target GPCRs [7,8].

Among the large family of GPCRs, there is a set of four receptors that share the physiological organic compound histamine as a ligand. They are termed H_1_R, H_2_R, H_3_R, and H_4_R, and they belong to the class-A of GPCRs [9]. Histamine plays a central role in the genesis of the symptoms observed in the context of allergic reactions, such as sneezing, pruritus, and excessive production of mucus [10]. The histamine H_1_ receptor is expressed in many different cell types including neurons, immune cells, vascular endothelial cells, and smooth muscle cells of respiratory or intestinal epithelium [11]. H_1_R plays an important role in type I hypersensitivity reactions, in which histamine is released from mast cells, binds to the receptor, and leads to its activation [12]. Due to its particular role for hypersensitivity reactions the histamine H_1_ receptor is one of the key targets in treating allergic reactions as well as sleeping disorders and emesis [5,11].

**Figure 1:**
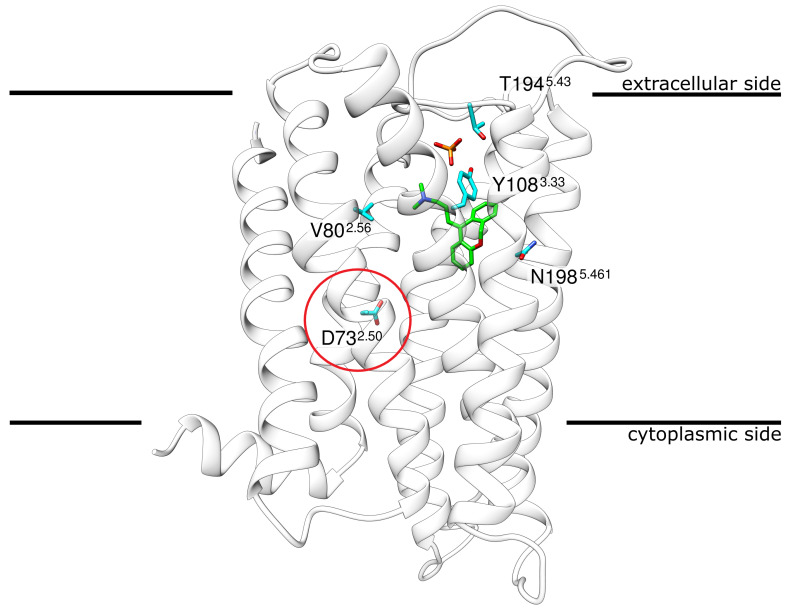
Structure of H_1_R in complex with the antagonist doxepin. H_1_R ribbon representation indicating the position of doxepin (green sticks) and phosphate (red/orange sticks) in the crystal structure. The encircled residue D73^2.50^ marks the approximate location at which a Na${}^{+}$ ion was found in other GCPRs. The four other residues shown in stick presentation represent the sites of mutation that were investigated in the present study.The membrane is schematically depicted as a black line. Coordinates from PDB entry 3RZE [15].

Doxepin, a tricyclic antihistamine, represents one of the strongest antihistamines [13,14] due to its low K_I_ of 2.2 nM [15]. Today, doxepin is mostly used in the treatment of major depressive disorders and insomnia as well as the treatment of atopic dermatitis or lichen simplex chronicus [16,17,18]. The structure of H_1_R, bound to the antagonist doxepin, has been available since 2011 [15] (Figure 1).

Although the H_1_R structure has already been exploited to study ligand binding [19,20,21,22] and to guide drug design [23,24,25], much less attention has been paid to the role of ions or H_1_R sequence variants observed in the human population as potential modulators of drug binding. As a rather unique feature not found in other GPCRs, the H_1_R crystal structure(PDB: 3RZE [15]) contains a phosphate ion bound on the extracellular side (Figure 1). H_1_R also contains anaspartate D73^2.50^ (position 2.50 according to Ballesteros–Weinstein nomenclature [26]), which is part of an allosteric sodium binding site in many class A GPCRs [27]. Sodium is known to represent a negative allosteric modulator of agonist binding in many GPCRs [27]. For human H_1_R, sodium dependency has also been reported for antagonist binding and most first-generation antihistamines exert a weaker effect in the presence of NaCl [28]. However, no sodium ion was detected in the vicinity of D73^2.50^ in the H_1_R crystal structure [15]. This raises the question of whether a sodium ion can actually be accommodated in this pocket and whether such a binding would affect doxepin interaction.

An additional factor which may affect ligand binding is the H_1_R sequence variants that are observed in a subset of the humanpopulation. Numerous variants were found in large-scale genome sequencing projects and are curated in the gnomAD database [29]. These variants represent the genetic variability in the human population, and most of them have not yet been studied to determine whether they are the cause of H_1_R-related diseases. Variants located in the vicinity of the ligand binding site are of particular interest because they may affect the binding of the physiological ligand or of drugs. One of these variants, T194A, has already been shown to reduce H_1_R affinity for the agonist histamine [30]. Cetirizine, an H_1_R antagonist, is bound with higher affinity and reduced stereoselectivity upon T194A mutation [30]. These effects prompted us to investigate sequence positions in the vicinity of the ligand binding site that display variability in the human population.From a search in the gnomAD database, we identified four naturally occurring sequence variants (V80I, Y108C, T194A, N198S) that are located in the vicinity of the doxepin binding site and might therefore affect antagonist binding.

In summary, the following investigations of H_1_R were performed in the present study: (i) identification of the energetically most favorable sodium binding site from metadynamics simulations, (ii) analysis of the dynamics of sodium and phosphate ions from 2-μs molecular dynamics (MD) simulations, and (iii) analysis of the effect of three types of potential modulators (sodium ions, phosphate ions, sequence variants) on doxepin binding affinity. Our study reveals a favorable binding site of sodium close to D73^2.50^, significant dynamics of the phosphate, and a relatively large effect of sequence variants compared to the ions on doxepin affinity. The implications of these findings for future directions of drug design are also discussed.

## 2. Results and Discussion

### 2.1. Determination of the Sodium Binding Site in H_1_R

The H_1_R contains the conserved aspartate (D73^2.50^) (red in Figure 1), which is part of an allosteric site that harbors a sodium ion in the inactive state of numerous GPCRs [27,31,32]. However, no sodium ion was detected in the H_1_R-doxepin complex structure.

To assess the sodium binding ability of H_1_R, we performed a multiple walker metadynamics simulation for the wildtype H_1_R in a similar fashion as done previously for the determination of the binding mode of orthosteric ligands [33].

**Figure 2:**
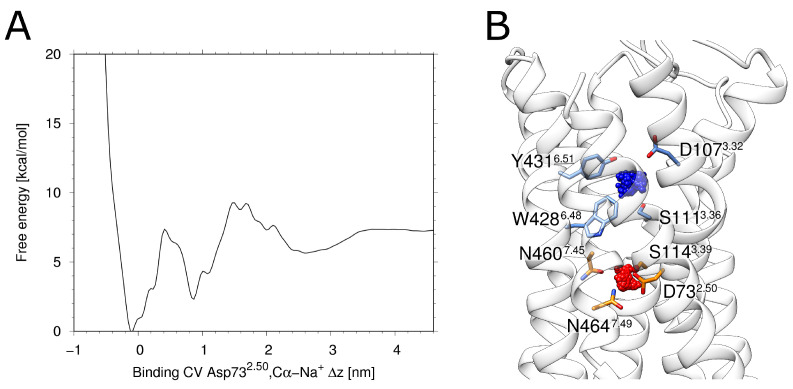
Metadynamics of the sodium ion. (**A**) Energy landscape obtained from the multiple walker metadynamics approach for H_1_R. Two distinct primary minima and one broader less-defined minimum can be seen. Panel (**B**) depicts the associated clusters. The most populated and therefore the lowest energy state is depicted in red, while the blue cluster represents the second distinct minimum from (**A**).

The free energy profile derived from the metadynamics simulation shows two distinct energy minima (Figure 2A). The deeper minimum at ~0 nm of the binding collective variable (CV) corresponds to a location of the allosteric pocket close to D73^2.50^(red in Figure 2B). The second minimum at a CV of ~1 nm (Figure 2A) corresponds to a location of the sodium ion within the orthosteric pocket (blue in Figure 2B). Such an energetic minimum has been previously observed in MD-simulations of the M_2_ muscarinic receptor and the δ-opioid receptor [34]. Like in H_1_R, the respective minimum is energetically less favorable compared to the minimum in the allosteric pocket and was described as a transient binding site [34]. The fact that the deepest minimum forH_1_R is observed in the vicinity of D73^2.50^ isalso in agreement with a previous accelerated MD study of Na${}^{+}$ binding [35] and thus supports that H_1_R uses the same preferred sodium binding site as other GPCRs.

### 2.2. Ion-Dependent Behavior of the H_1_R

Our study aimed to assess the dynamics of the three ligands doxepin, sodium and phosphate. For that purpose, three different H_1_R complexes were modeled that either contained doxepin (D), sodium (S) or phosphate (P). These systems were termed H_1_R-D, H_1_R-S and H_1_R-P, respectively. To assess the existence of cooperative effects between different ligands, we also generated one system containing doxepin and sodium (H_1_R-DS) and another system with all three ligands (H_1_R-DSP).

**Figure 3:**
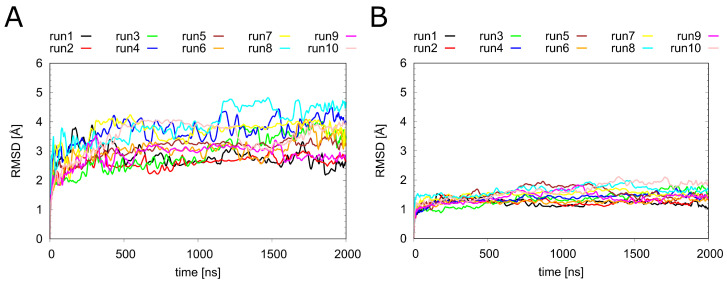
Dynamics of H_1_R in the presence of different ligands. Root-mean-square deviation (RMSD) calculated for (**A**) the entire backbone and (**B**) only the Cα atoms of the transmembrane helices of the H_1_ receptor. The individual runs denoted above the graphs correspond to the following systems: H_1_R-DS (run1, run2), H_1_R-DSP (run3, run4). H_1_R-D (run5, run6), H_1_R-P (run7, run8) and H_1_R-S (run9, run10).

Before looking at the individual ligands in detail, we analyzed the overall dynamics of H_1_R (Figure 3). The RMSD values show that all systems remained stable over the simulation time of 2 μs. Notably, the overall dynamics of H_1_R were not significantly affected by the presence or absence of the ligands investigated in the present study. To understand the local effects of the ligands in more detail, they were analyzed separately, as described in the sections below.

### 2.3. Dynamics of the Sodium Ion

**Figure 4:**
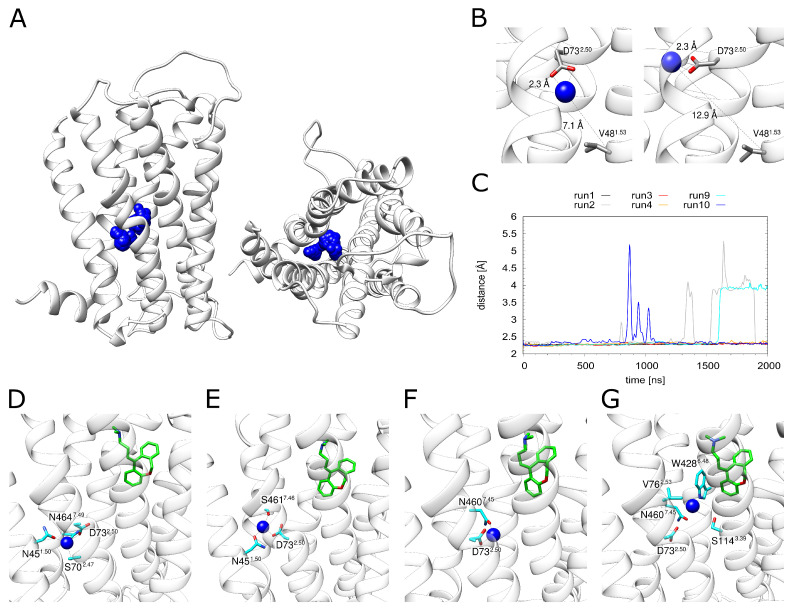
Dynamics of the sodium ion. (**A**) H_1_R structure (white ribbons) depicting the Na+ position (blue spheres) derived from simulation at time intervals of 1 ns. (**B**) The Na+ ion can adopt different positions in the binding pocket while still remaining attached to D73^2.50^. (**C**) Distance between D73^2.50^ and the sodium ion for all simulations that contained a sodium ion. H_1_R-DS (run1, run2), H_1_R-DSP (run3, run4) and H_1_R-S (run9, run10). (**D**–**G**) Frames from the H_1_R-DS (run2) simulation at 0 ns, 100 ns, 1000 ns and 1700 ns. Sodium is depicted as a blue sphere, interacting residues are shown in cyan and the ligand doxepin is highlighted by green highlighted carbon atoms. The receptor is shown as a white ribbon.

Monitoring the dynamics of Na${}^{+}$ in the allosteric pocket revealed rather large fluctuations of the ion within the pocket (Figure 4A). A more detailed inspection (Figure 4B) showed that the Na${}^{+}$ can sample different positions while remaining attached to D73^2.50^ (as exemplified in the change of the distance to V48^1.53^ in Figure 4B). These motions are accompanied by a rotation of the D73^2.50^ side chain.

D73^2.50^ represents the major interaction partner of Na${}^{+}$ (Figure 4C). Other polar residues involved in Na${}^{+}$ coordination include N45^1.50^, S70^2.47^, N460^7.45^, S461^7.46^ and N464^7.49^. However, the strength and pattern of the interaction with these residues varies significantly between the individual simulation runs (Figure S1). This situation is analyzed in detail for H_1_R-DSP with bound doxepin in Figure 4D–G. The Na${}^{+}$ moves within the allosteric pocket. The largest motion is observed towards W428^6.48^, i.e., in the direction of the orthosteric pocket (Figure 4G), which is accompanied by a detachment of the Na${}^{+}$ from D73^2.50^ (Figure 4C). The same type of motion is also detected in the absence of doxepin (see H_1_R-S in Figure 4C). However, no complete movement into the orthosteric pocket is observed on the timescale of our simulations regardless of doxepin being present or not (Figure 4C).

The distance between the energetically favorable Na${}^{+}$ positions in two pockets is ~10 Å, which can be seen in the energy landscape plot in Figure 2A. The maximal movement of the Na${}^{+}$ ion from the allosteric towards the orthosteric pocket is ~5.5 Å (Figure 4C). This distance corresponds almost exactly to the position of the local maximum (energy barrier) separating the allosteric and orthosteric pocket (Figure 2A). This is in line with the observation that the allosteric pocket represents the energetically most favorable site for accommodating a Na${}^{+}$ and with the presence of an energetic barrier between the two minima found in the orthosteric and allosteric pocket (Figure 2A). Due to this energetic barrier, a transition of Na${}^{+}$ ions is expected to represent a rare event that may only occur on timescales longer than those of the present simulations.

### 2.4. Dynamics of the Phosphate Ion

**Figure 5:**
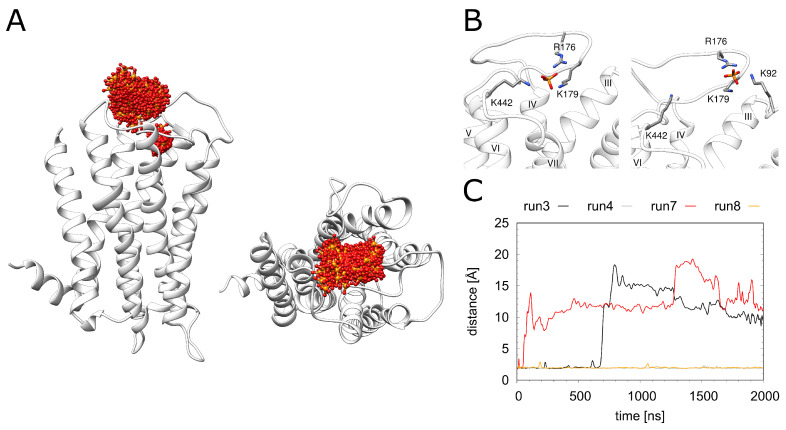
Dynamics of the phosphate ion. (**A**) H_1_R structure (white ribbon) depicting the phosphate position (red/orange sticks) derived from simulation at time intervals of 1 ns. (**B**) Two representative phosphate binding modes and interacting residues. (**C**) Distance between K191^5.40^ and the phosphate for the H_1_R-DSP (run3, run4) and H_1_R-P (run7, run 8) simulations.

As a structural feature that was not observed in related GPCRs, H_1_R contains a phosphate ion bound in the vicinity of the orthosteric ligand doxepin (Figure 1). In the crystal structure, major interactions with phosphate are formed by the 2 lysines (K179 and K191^5.40^) and 1 histidine (H450^7.34^). In the MD simulation, the phosphate ion exhibits a rather high mobility (Figure 5A); however, no dissociation from H_1_R is observed on the timescale of the simulations. During the entire simulation time, the phosphate remains attached to K179, whereas the interaction with K191^5.40^ may be replaced by interactions with other basic residues of the extracellular region (K92^23.49^, R176, K442) (Figure S2).The interactions between H450^7.34^ and the phosphate are immediately lost in three of the four simulation runs. Weak transient interactions are observed between H450^7.34^ and the phosphate only in one run (Figure S2D). Two snapshots of alternative phosphate interaction patterns observed in the simulations are depicted in Figure 5B. Figure 5C shows that the interaction with K191^5.40^ is lost in 2 out of the 4 simulations. Interestingly, the pattern of the observed interactions does not correlate with the presence or absence of doxepin indicating that these two sites are not tightly coupled.

### 2.5. Dynamics and Energetics of Doxepin Binding

**Figure 6:**
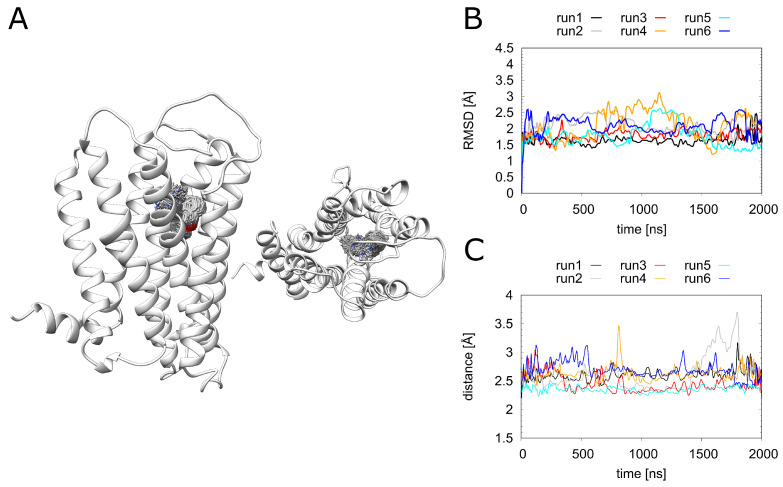
Doxepin interactions. (**A**) H_1_R structure (white ribbon) depicting the doxepin position derived from simulation at time intervals of 1 ns. Doxepin is shown as gray sticks with oxygen in red and nitrogen in blue. (**B**) The ligand RMSD and (**C**) minimum doxepin-D107^2.50^ distance over the simulation time. H_1_R-DS (run1, run2), H_1_R-DSP (run3, run4), H_1_R-D (run5, run6).

During the MD simulations, doxepin remains stably bound in the pocket (Figure 6A). There are only small fluctuations of the RMSD over the simulation time (Figure 6B), and the key electrostatic interaction between doxepin and D107^3.32^ remains intact for more than 90% of the simulation time (Figure 6C). Detailed information regarding residues involved in doxepin binding is given in the supplement (Figure S3). The observed fluctuations are rather similar for all simulations (Figure 6B), showing that the stability of the doxepin binding mode is not significantly affected by the presence of a phosphate ion or a Na${}^{+}$ ion in the allosteric pocket. We did not investigate whether the binding of doxepin and Na${}^{+}$ in the orthosteric pocket at the same time would be feasible. In order to quantify the effects of these ions on the doxepin binding affinity, we performed an MM/GBSA analysis (Table 1).

The doxepin binding energy in the absence of additional ions (H_1_R-D system) is 0.7 to 0.8 kcal·mol^−1^ more favorable compared to the H_1_R-DS or H_1_R-DSP systems. The highly similar interaction energy between the latter two systems suggests that the presence of sodium ions is the key effect responsible for the weaker interaction, whereas the additional presence of the phosphate in H_1_R-SP does not cause any significant changes. These observations are also in agreement with the experimental data from the literature for H_1_R: Hishinuma et al. [28] detected a 3.7-fold weaker binding for doxepin in the presence of 100 mM NaCl compared to the absence of NaCl. With respect to phosphate, Shimamura et al. [15] showed that the type of buffer used (PBS vs. HEPES) does not significantly affect doxepin binding affinity.

### 2.6. Effect of H_1_R Sequence Variants on Doxepin Affinity

The following investigation aimed to assess the effect of four sequence variants (V80I, Y108C, T194A, N198S) in the human population that are located in the vicinity of the doxepin binding site and might therefore affect antagonist binding. The mutations were modeled in the context of the H_1_R-DS, and their effect on binding affinity was assessed from 500 ns simulations (Table 2).

**Figure 7:**
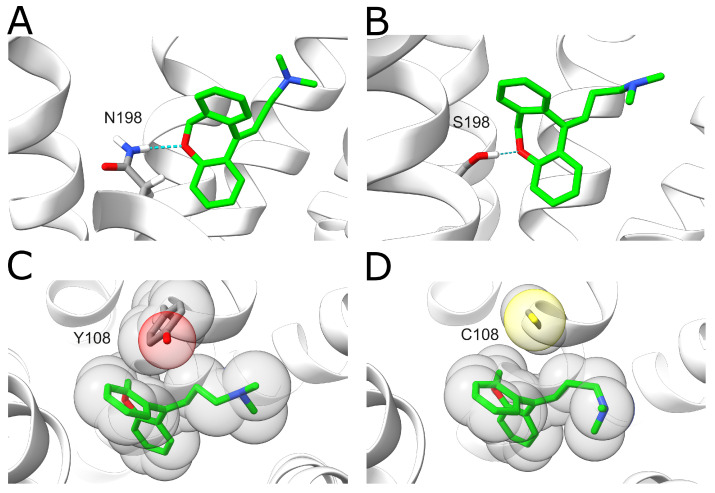
Effect of H_1_R sequence variants N198S and Y108C on doxepin binding. (**A**) A hydrogen bond (blue dotted line) is formed between N198^5.461^ and doxepin. N198^5.461^ and doxepin are shown in stick representation and colored according to the type of element (carbons of N198^5.461^ and doxepin are colored in gray and green, respectively). (**B**) In the N198S variant, a weaker hydrogen bond (thin dotted line) is formed due to the shorter serine sidechain. (**C**) Interaction between Y108^3.33^ and doxepin. The interaction partners are shown in stick representation, and the volume of the atoms is additionally indicated as transparent spheres. (**D**) The smaller sidechain of the Y108C variant results in a loss of hydrophobic interactions with doxepin.

The results show that these variants display decreased doxepin binding affinities compared to the wildtype with a change of 1–2 kcal·mol^−1^. The largest effect is observed for the N198S variant. In the wildtype, N198^5.461^ forms a hydrogen bond with the oxygen of the central doxepin ring (Figure 7A). This hydrogen bond is formed in 59.0% of the analyzed snapshots for the wildtype, whereas it is only formed for 19.8% of the snapshots in the N198S mutant. This difference can most likely be attributed to the fact that the serine sidechain is shorter compared to asparagine, resulting in larger distances to the doxepin ligand (Figure 7B). A large effect is also observed for the Y108C variant. In the wildtype, the Y108^3.33^ ring forms tight hydrophobic interactions with the doxepin ring system (Figure 7C), which cannot be formed by the much smaller non-aromatic cysteine side chain (Figure 7D).

## 3. Materials and Methods

### 3.1. Molecular Dynamics Simulations

The crystal structure of the human histamine H_1_ receptor in complex with the antagonist doxepin (PDB code 3RZE [15]) was used as template for the system generation. The T4 lysozyme used for crystallization was removed, and the resulting gap between C221^5.69^ and L405^6.25^ was closed by an 8-residue spacer (sequence GSGSGSGS) using Modeller 9.16 [36]. The systems defined for the investigation of ions and sequence variants are described in Table 3. All simulations were carried out with gromacs19 [37]. We used the force fields ff99SB for the proteins, and gaff for the DOPC molecules as well as for the ligand doxepin [38]. Water was simulated using the SPC model. Each system was minimized and equilibrated applying a previously established protocol [33]. The minimization consisted of three consecutive steps with restraints applied to different subsets of atoms (first to all atoms except for water molecules, second only to Cα atoms and third without any restraints). During minimization, 2500 steps of the steepest descent algorithm followed by 2500 steps of the conjugate gradient algorithm were applied. A harmonic potential with a force constant of 10 kcal·mol${}^{-1}\cdot$Å${}^{-2}$ was used for the atom restraints. The membrane equilibration was performed in 300 consecutive simulations, 100 ps each, during which water molecules inherently diffusing into the membrane were deleted using an in-house Perl script while the receptor and ligand atoms were restrained with a force constant of 5 kcal·mol${}^{-1}\cdot$Å${}^{-2}$. The temperature was constantly kept at 310 K by a Berendsen thermostat [39]. Surface-tension coupling with a reference *z* pressure of 1 bar and a reference surface tension of 1.1 nm·bar were applied. The SHAKE algorithm [40] allowed for a 2 fs time-step during equilibration and production runs. Periodic boundary conditions were set for the *x*, *y* and *z* direction. A summary of all simulation runs performed is given in Table 3.

### 3.2. Metadynamics

The metadynamics simulation strategy used for the determination of the sodium binding mode was adopted from a previous work of Söldner et al. [33] and Saleh et al. [41]. For all metadynamics simulations, we used Gromacs 2016.3 in combination with the plumed 2.3.1 [42] plugin. A well-tempered metadynamics was set up using the z component of the distances between the Cα atom of D73^2.50^ and the sodium ion as the collective variable (CV). For limiting the sampled area, lower and upper walls $z_{low}$ and $z_{up}$ were chosen at positions of −0.5 nm and 5.2 nm as CV boundary conditions. In order to further limit the exploration of the bulk solvent, a bell-shaped funnel was used. The initial metadynamics simulations, which aimed at mapping the sodium binding pathway, were run with a bias height of 7 kJ·mol^−1^, a Gaussian width of 0.1 and a bias factor of 50. The subsequent simulations using the multiple walker setup were carried out with a bias factor of 20 and an initial bias of 5 kJ· mol^−1^.

### 3.3. Trajectory Analysis

Cpptraj from AmberTools 18 [43] was the main tool used for post-processing and analyzing trajectories. Contacts were calculated with the nativecontacts command using 5 Å as distance criteria. The mm_pbsa.pl script for the MM/GBSA method was applied for calculating interaction energies between the ligand and receptor with default parameters [44,45,46]. The presence of hydrogen bonds was investigated using VMD [47] using the snapshots from the second half of the simulation. UCSF Chimera [48] and ChimeraX [49] were used for structure visualization. Gnuplot [50] was used for plotting graphs.

## 4. Conclusions

The present study has investigated the influence of ions and mutations on the affinity of H_1_R for the antagonist doxepin. Although the present study shows that the presence of sodium or phosphate does not significantly affect doxepin binding affinity, the interaction patterns of these ions may nevertheless provide valuable information for the design of more affine or more specific drugs. Such information has, for example, already been exploited for the design of second-generation antihistamines carrying a carboxyl group that was designed to interact with the phosphate binding site, thereby enhancing specificity for H_1_R binding [15]. The present study provides information about the relative stability of interactions between positively charged lysines in H_1_R and the anionic phosphate group. Performing similar simulations for second-generation histamines may help to optimize the stability of contacts with acidic groups by variation in the drug scaffold.

Our simulations also confirmed that H_1_R contains a sodium binding site in the allosteric pocket that can accommodate a sodium ion and gave detailed information about the residues preferentially interacting with Na+. This data may also be exploited in the future to design H_1_R ligands that target the conserved sodium binding pocket. Such an approach has already successfully been applied in case of the leukotriene B4 receptor BLT1 by designing the bitopic ligand BIIL260 that spans from the orthosteric pocket to the sodium-binding residues D^2.50^ [51]. Generally, the investigated sequence variants have a larger effect on doxepin binding affinity compared to sodium and phosphate ions. Although sequence variants in the immediate vicinity of the ligand binding site are rare, they might affect the potency of H_1_R targeting drugs. Similar effects have been shown previously for other GPCRs, e.g., experiments have demonstrated that certain variants of µ-opioid and cholecystokinin-A receptors lead to altered or adverse drug response [52]. These findings suggest that personalized medical treatments, which consider individual mutation patterns, could increase the efficiency of GPCR-targeting drugs.

## Figures and Tables

**Table 1:** Interaction energies between doxepin and the H_1_ receptor. Averages and standard errors of mean (n=10,000 frames from the second half of the simulations) were calculated for the binding energies using MM/GBSA. Values are averaged over two independent simulation runs for each system.

System	Binding Energy [kcal·mol^−1^]
H_1_R-D	−34.52 ± 0.04
H_1_R-DS	−33.71 ± 0.05
H_1_R-DSP	−33.81 ± 0.03

**Table 2:** Interaction energies between doxepin and the H_1_ receptor. Averages and standard deviations (n=5000 frames) were calculated using MM/GBSA. Values are averaged over two independent simulation runs for each system.

System	Binding Energy [kcal·mol^−1^]
H_1_R-wildtype	−33.71 ± 0.05
H_1_R-N198S	−31.34 ± 0.04
H_1_R-T194A	−32.74 ± 0.04
H_1_R-Y108C	−31.75 ± 0.05
H_1_R-V80I	−32.27 ± 0.04

**Table 3:** Overview of the simulations performed. The table lists all MD simulations conducted and the respective names by which they are referred to in the figures and manuscript text. Further, the number of runs and the time simulated are given. The following columns describe the composition of the respective systems, i.e., whether the histamine H_1_ receptor (H_1_R), the ligand doxepin, the phosphate ion (PO${}_{4}^{3-}$), or the sodium ion (Na${}^{+}$) was present. For the simulations of mutant H_1_R, the type of mutation is indicated in the first column. The mutation runs are based on the setup for run1 and therefore bear the same overall molecule counts. The starting conformations are taken from the last 100 ns time frames of run 1.

Simulation	Abbreviation	Runs × Time	H_1_R	Doxepin	PO ${}_{4}^{3-}$	Na ${}^{+}$	♯Atoms
H_1_R-DS	run1 & run2	2 × 2 μs	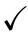	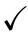	×	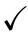	125,376
H_1_R-DSP	run3 & run4	2 × 2 μs	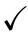	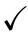	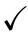	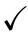	125,525
H_1_R-D	run5 & run6	2 × 2 μs	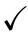	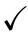	×	×	125,374
H_1_R-P	run7 & run8	2 × 2 μs	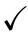	×	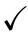	×	124,968
H_1_R-S	run9 & run10	2 × 2 μs		×	×		125,367
H_1_R-N198S	mut1 & mut2	2 × 0.5 μs	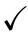	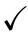	×	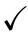	125,307
H_1_R-T194A	mut3 & mut4	2 × 0.5 μs	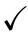	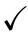	×	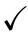	125,087
H_1_R-Y108C	mut5 & mut6	2 × 0.5 μs	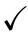	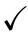	×	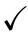	125,276
H_1_R-V80I	mut7 & mut8	2 × 0.5 μs			×		125,049

## Data Availability

Not applicable.

## Supplementary Materials

The following are available online at https://www.mdpi.com/article/10.3390/ijms23031420/s1.

## Abbreviations

The following abbreviations are used in this manuscript:

GPCR	G protein-coupled receptor
H_1_R	Histamine H_1_ receptor
D	Doxepin
S	Sodium
P	Phosphate
RMSD	Root-mean-square deviation
CV	Collective variable
MD	Molecular dynamics simulation
PDB	Protein data bank
DOPC	Dipalmitoylphosphatidylcholin
