# Supplementary material for: Effect of Ions and Sequence Variants on the Antagonist Binding Properties of the Histamine H1 Receptor
Source: Int J Mol Sci. 2022 Jan 26;23(3):1420. doi: 10.3390/ijms23031420 (PMC8836275; doi:10.3390/ijms23031420)
Supplement: Supplementary file 1 [file ijms-23-01420-s001.zip › supplement.pdf]

Supplementary Material: Effect of ions and sequence variants on the antagonist binding properties of the histamine H_1 receptor

Marcus Conrad [1], Christian A. Söldner [1] and Heinrich Sticht [1,2]*

A

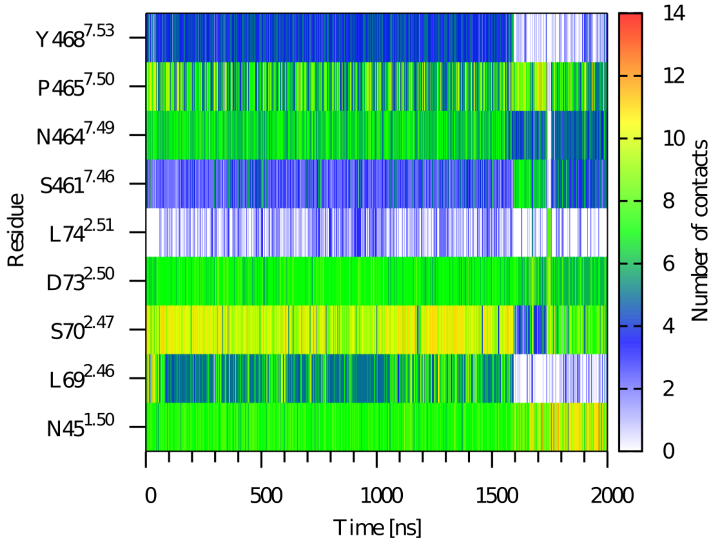

B

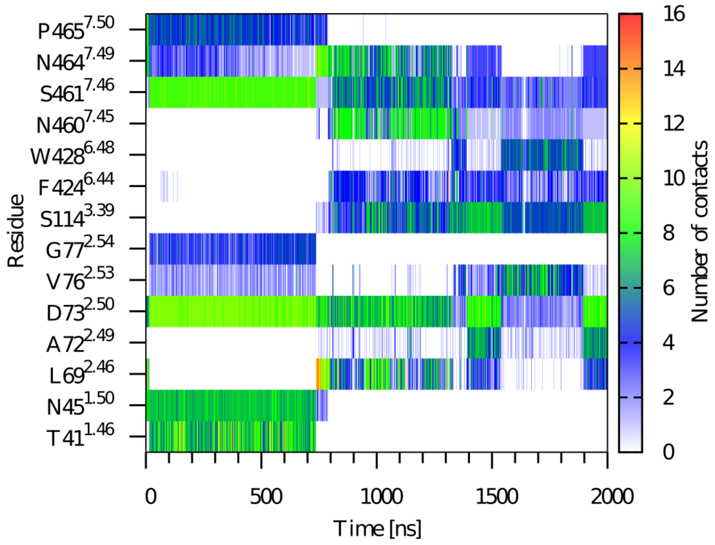

C

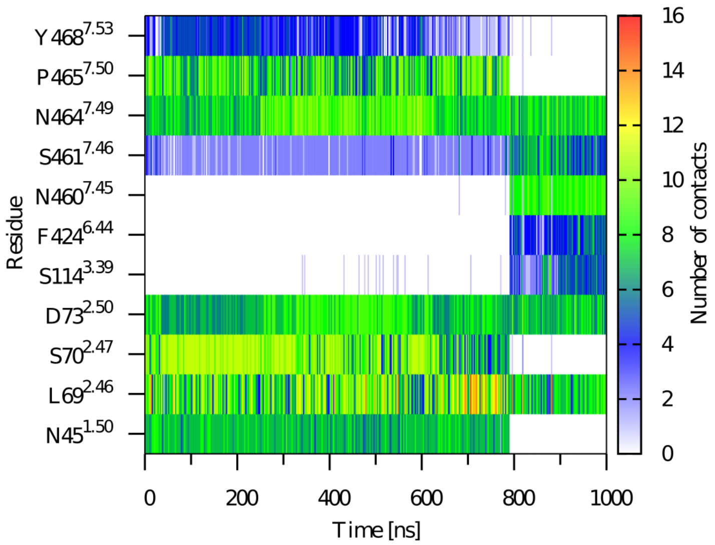
Y468 7.53
P465 7.50
N464 7.49
S461 7.46
N460 7.45
F424 6.44
S114 3.39
D73 2.50
S70 2.47
L69 2.46
N45 1.50
Residue
0
200
400
600
800
1000
Time [ns]
16
14
12
10
8
6
4
2
0
Number of contacts

D

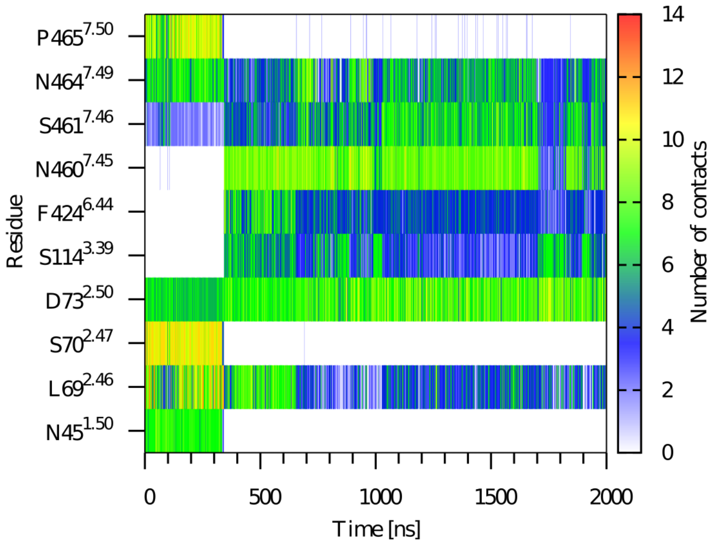
P465 7.50
N464 7.49
S461 7.46
N460 7.45
F424 6.44
S114 3.39
D73 2.50
S70 2.47
L69 2.46
N45 1.50
Residue
0
500
1000
1500
2000
Time [ns]
14
12
10
8
6
4
2
0
Number of contacts

E

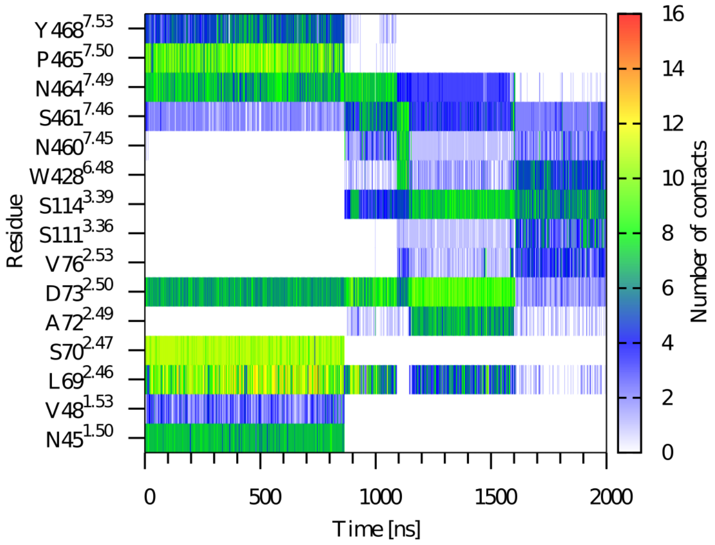

F

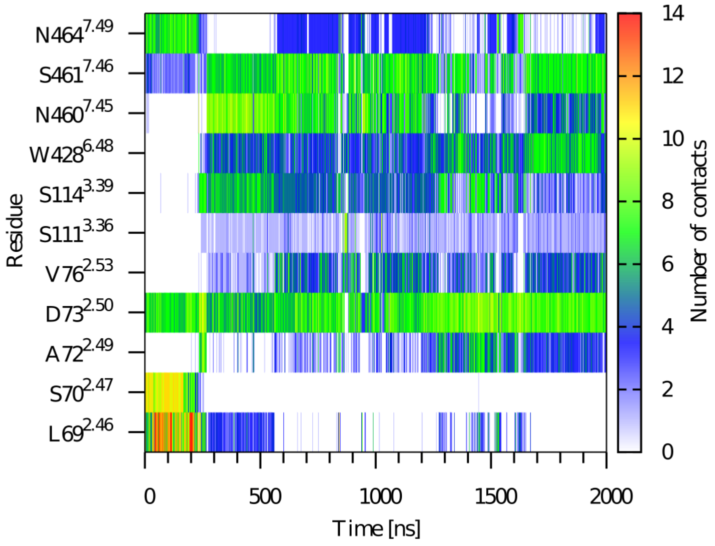

Figure S1. Sodium Interactions Number of contacts between Na+ and H_1R over the simulation time. The simulation time is shown as horizontal axis and the interacting H_1R residues on the vertical axis. The number of contacts is color coded according to the scale shown on the right side of each panel. The Panels A-F represent the runs 1-4,9,10 found in Table 3 (main document).

A

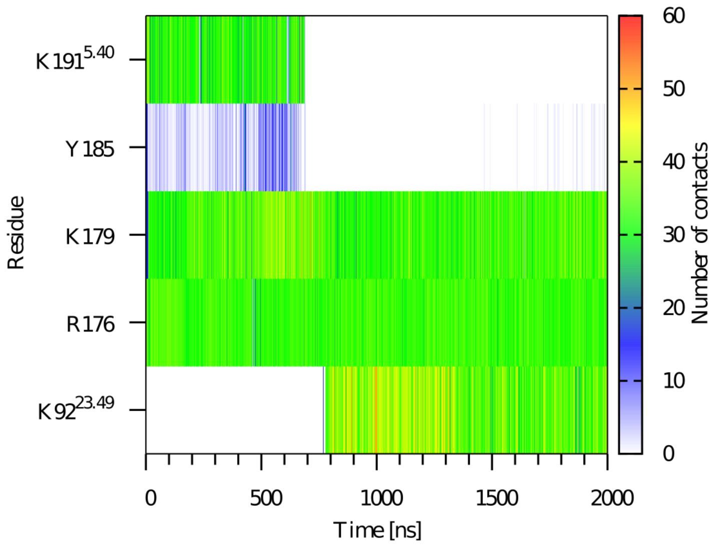
K191$^{5.40}$
Y185
K179
R176
K92$^{23.49}$
Residue
0
500
1000
1500
2000
Time [ns]
60
50
40
30
20
10
0
Number of contacts

B

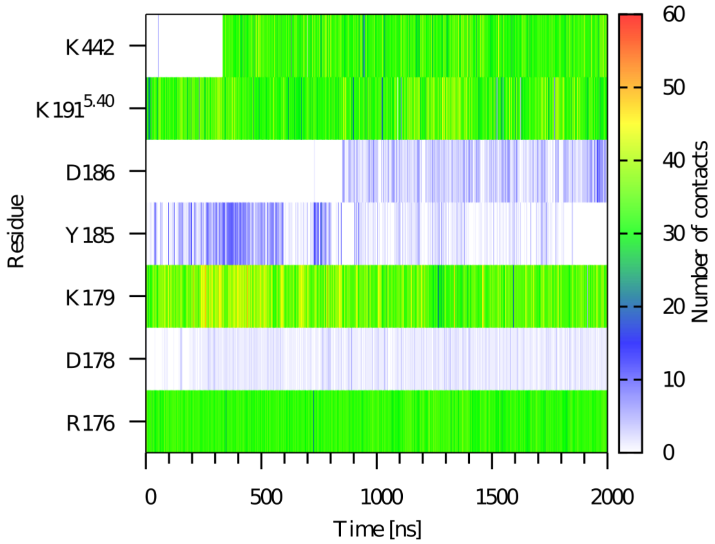
K442
K191$^{5.40}$
D186
Y185
K179
D178
R176
Residue
0
500
1000
1500
2000
Time [ns]
60
50
40
30
20
10
0
Number of contacts

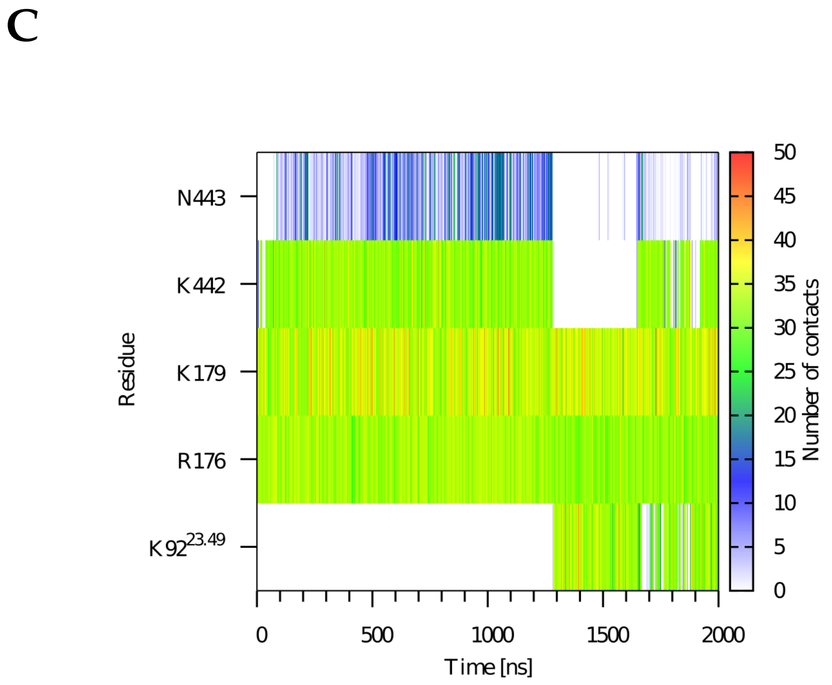

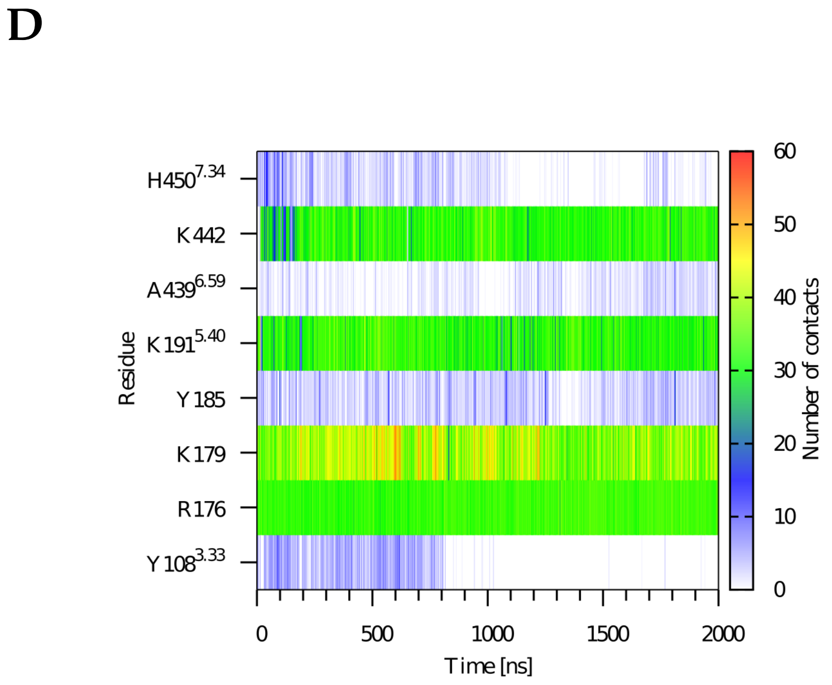

Figure S2. Phosphate Interactions Number of contacts between phosphate ion and H_1R over the simulation time. The simulation time is shown as horizontal axis and the interacting H_1R residues on the vertical axis. The number of contacts is color coded according to the scale shown on the right side of each panel. The Panels A-D represent the runs 3,4,7,8 found in Table 3 (main document).

A

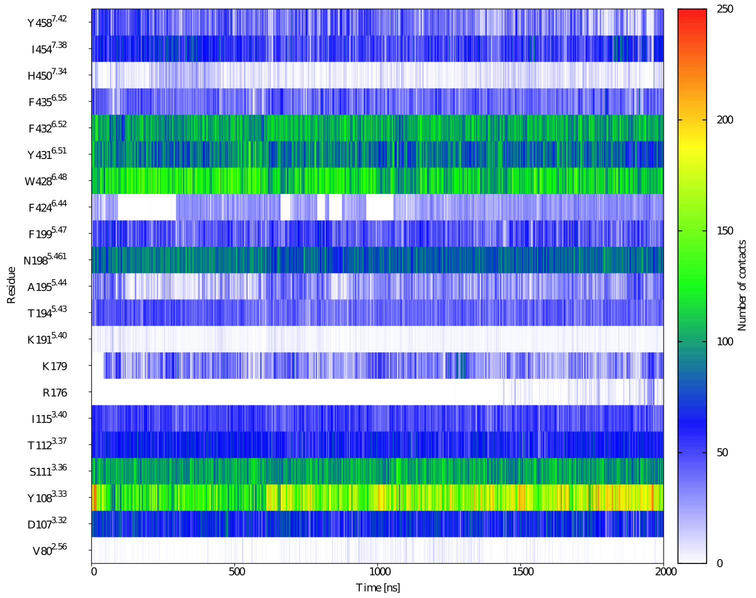

Y458 7.42
I454 7.38
H450 7.34
F435 6.55
F432 6.52
Y431 6.51
W428 6.48
F424 6.44
F199 5.47
N198 5.461
A195 5.44
T194 5.43
K191 5.40
K179
R176
I115 3.40
T112 3.37
S111 3.36
Y108 3.33
D107 3.32
V80 2.56
Residue
0
500
1000
1500
2000
Time [ns]
250
200
150
100
50
0
Number of contacts

B

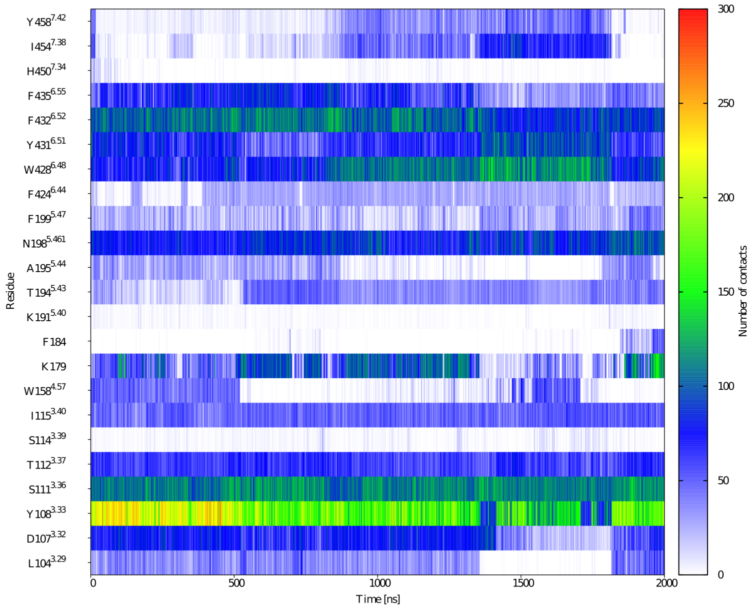

Y458 7.42
I454 7.38
H450 7.34
F435 6.55
F432 6.52
Y431 6.51
W428 6.48
F424 6.44
F199 5.47
N198 5.461
A195 5.44
T194 5.43
K191 5.40
F184
K179
W158 4.57
I115 3.40
S114 3.39
T112 3.37
S111 3.36
Y108 3.33
D107 3.32
L104 3.29
Residue
0
500
1000
1500
2000
Time [ns]
300
250
200
150
100
50
0
Number of contacts

C

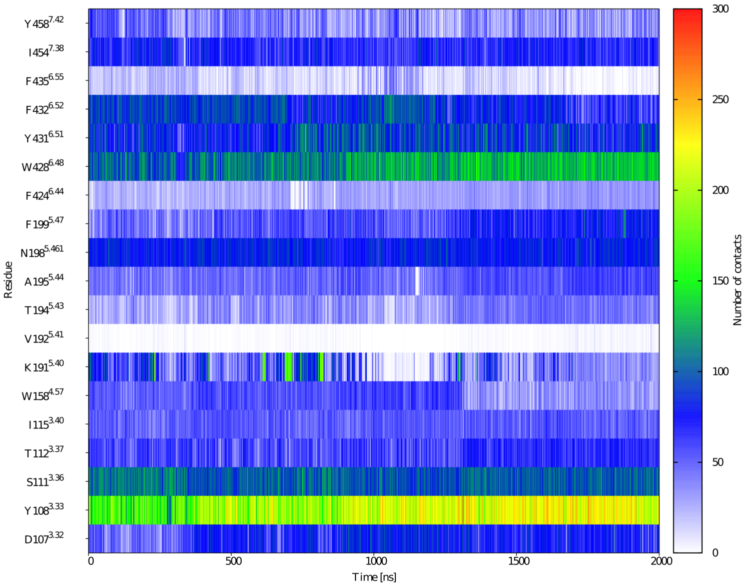
Y 458$^{7.42}$
I 454$^{7.38}$
F 435$^{6.55}$
F 432$^{6.52}$
Y 431$^{6.51}$
W428$^{6.48}$
F 424$^{6.44}$
F 199$^{5.47}$
N198$^{5.461}$
A 195$^{5.44}$
T 194$^{5.43}$
V 192$^{5.41}$
K 191$^{5.40}$
W158$^{4.57}$
I 115$^{3.40}$
T 112$^{3.37}$
S111$^{3.36}$
Y 108$^{3.33}$
D107$^{3.32}$
Residue
0
500
1000
1500
2000
Time [ns]
300
250
200
150
100
50
0
Number of contacts

D

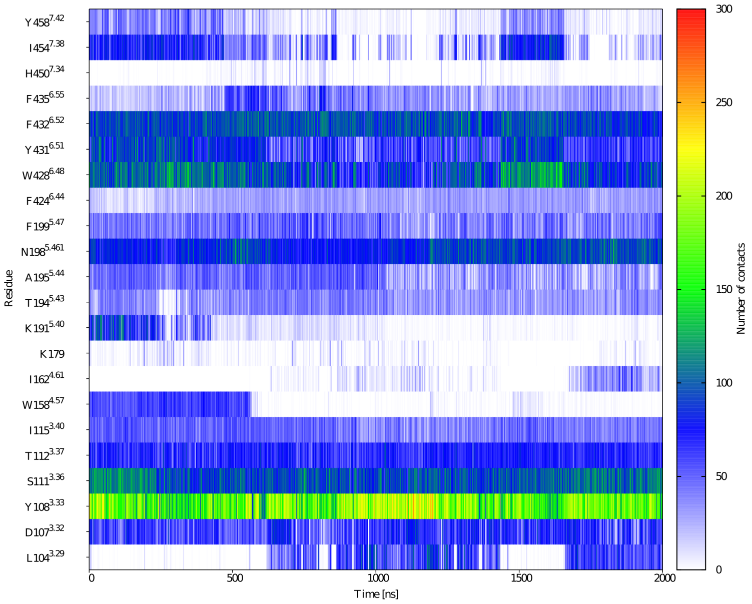
Y 458$^{7.42}$
I 454$^{7.38}$
H450$^{7.34}$
F 435$^{6.55}$
F 432$^{6.52}$
Y 431$^{6.51}$
W428$^{6.48}$
F 424$^{6.44}$
F 199$^{5.47}$
N198$^{5.461}$
A 195$^{5.44}$
T 194$^{5.43}$
K 191$^{5.40}$
K 179
I 162$^{4.61}$
W158$^{4.57}$
I 115$^{3.40}$
T 112$^{3.37}$
S111$^{3.36}$
Y 108$^{3.33}$
D107$^{3.32}$
L104$^{3.29}$
Residue
0
500
1000
1500
2000
Time [ns]
300
250
200
150
100
50
0
Number of contacts

E

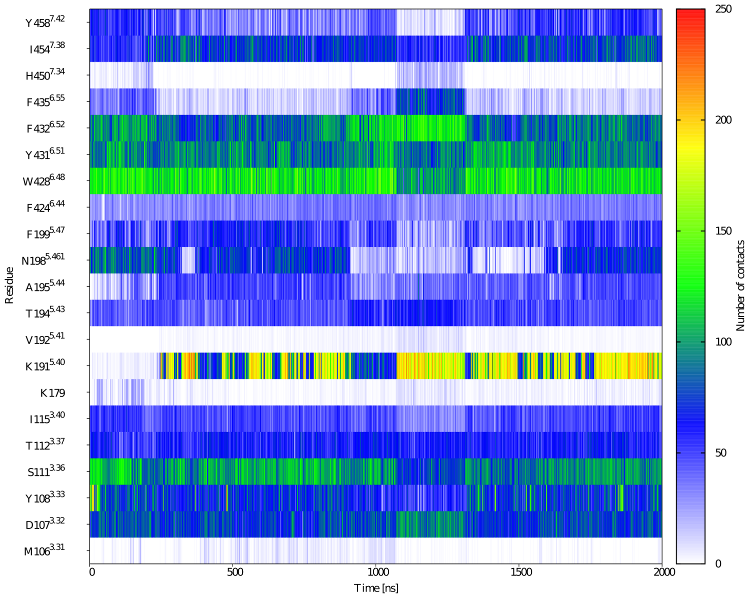

F

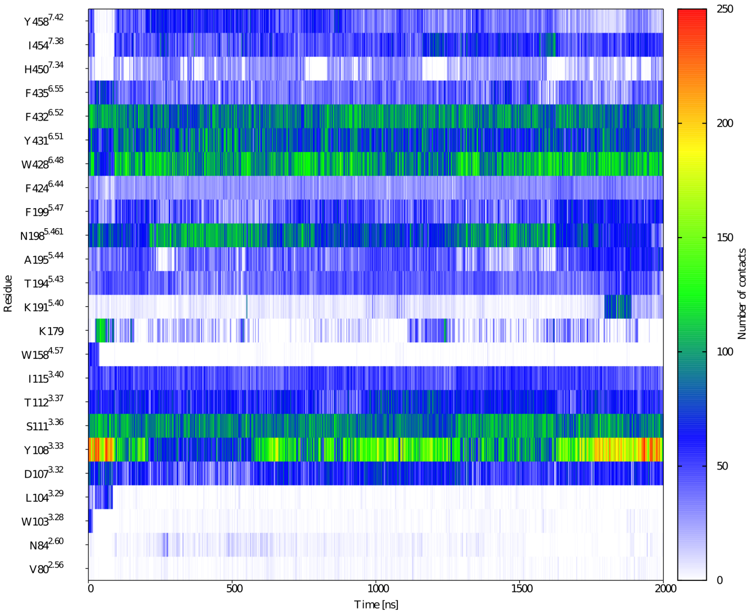

Figure S3. Doxepin Interactions Number of contacts between doxepin and H_1R over the simulation time. The simulation time is shown as horizontal axis and the interacting H_1R residues on the vertical axis. The number of contacts is color coded according to the scale shown on the right side of each panel. The Panels A-F represent the runs1-6 found in Table 3 (main document).
